# Electronic and Magnetic Properties of Stone–Wales Defected Graphene Decorated with the Half-Metallocene of *M* (*M* = Fe, Co, Ni): A First Principle Study

**DOI:** 10.3390/nano8070552

**Published:** 2018-07-20

**Authors:** Kefeng Xie, Qiangqiang Jia, Xiangtai Zhang, Li Fu, Guohu Zhao

**Affiliations:** 1 Provincical Key Laboratory of Gansu Higher Education for City Environmental Pollution Control, School of Chemistry and Chemical Engineering, Lanzhou City University, Lanzhou 730070, China; 2 State Key Laboratory of Plateau Ecology and Agriculture, Qinghai University, Xining 810016, China; 2015990037@qhu.edu.cn (Q.J.); 2017990017@qhu.edu.cn (X.Z.); 3 Collage of Materials and Environmental Engineering, Hangzhou Dianzi University, Hangzhou 310018, China; fuli@hdu.edu.cn

**Keywords:** Stone–Wales defected graphene, half-metallocene, adsorption energy, density of states, and magnetic property

## Abstract

The geometrical, electronic structure, and magnetic properties of the half-metallocene of *M* (*M* = Fe, Co, Ni) adsorbed on Stone–Wales defected graphene (SWG) were studied using the density functional theory (DFT), aiming to tune the band structure of SWG. The introduction of cyclopentadienyl (Cp) and half-metallocene strongly affected the band structure of SWG. The magnetic properties of the complex systems originated from the 3D orbitals of *M* (*M* = Fe, Co, Ni), the molecular orbital of Cp, and SWG. This phenomenon was different from that found in a previous study, which was due to metal ion-induced sandwich complexes. The results have potential applications in the design of electronic devices based on SWG.

## 1. Introduction

Graphene, which is as a typical two-dimensional (2D) material, has aroused considerable attention because of its special properties and promising potential applications in electronic devices, nanocomposites, molecule sensors, transparent electrodes in light emitting diodes (LED), and photovoltaic devices [1,2,3,4]. The typical structural defects [5] in graphene are vacancies [6], impurities [7,8,9,10], Stone–Wales (SW) defects [11,12,13,14], and pentagonal–octagonal defects [15,16]. The various defects of graphene can alter its electronic and mechanical properties significantly [5,17,18]. The most common topological defect in graphene is SW defect, which is formed by an in-plane 90° rotation of a C-C bond in a hexagon ring with fixing the middle point of this bond. SW defects in graphene have been studied widely because the opening band gap of electronic structure is applied to tune the band structure in the design of electronic devices [14,19,20,21]. SW defect is a classical topological defect in graphene and Stone-Wales graphene (SWG) includes two pairs of pentagonal–heptagonal rings. Compared to perfect graphene, SW graphene is more sensitive in absorbing mercaptan, ozone, and formaldehyde [22,23]. The interactions between graphene and different molecules are important for sensor devices based on the graphene. Therefore, experimental or theoretical research has focused on understanding the effect of the electronic and magnetic properties of graphene when molecules adsorb on graphene [24,25,26,27]. Transition metals (TMs) absorbed in carbon nanotube and graphene have been given great attention because the absorbed TM atoms can generate novel physical, chemical, and mechanical properties [28,29,30].

In this study, we used first principle calculations to explore the effect of interactions between SWG and cyclopentadienyl (Cp) or half-metallocene of *M* (*M* = Fe, Co, Ni) on their electronic and magnetic properties. Moreover, the geometry and electronic structures were investigated.

## 2. Calculation Details

**Figure 1:**
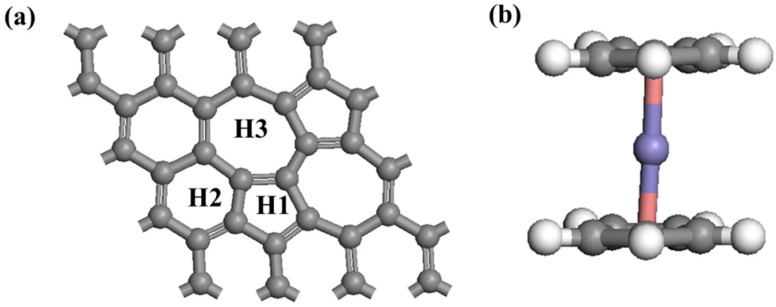
Optimized atomic structures of (**a**) Stone-Wales graphene (SWG) and (**b**) ferrocene.

Calculations were performed using DFT with van der Waals correctionsas implemented in the CASTEP software of in Material Studio. Generalized gradient approximation (GGA) with the Perdew–Burke–Ernzerhof (PBE) exchange correlation function was used [31]. The SW graphene slab was a 3 × 3 × 1 supercell (9.84 × 9.84 × 15.00 Å, 32 C atoms). The cutoff energy was set to 300 eV. K point of the Brillouin zone and was sampled using 5 × 5 × 1 [32]. The energy convergence standard was 10^–5^ eV per atom during all structural relaxation. The forces on relaxation were less than 0.05 eV/Å. Test calculations which used a higher cutoff energy (400 eV) or a larger K point (7 × 7 × 1) between the SWG sheets were performed and showed less than 4% improvement to the simulation accuracy. Therefore, the calculation parameters were considered to be accurate. We have considered the van der Waals interaction [24,25] between the SWG and Cp. A Cp or half-metallocene was located at the center of the carbon ring of SWG. Considering the structure symmetry of SWG, the hollow sites included the centers of a pentagon ring (H1), hexagon ring (H2), and heptagon ring (H3) (Figure 1).

The adsorption system stability was estimated using the absorption energy $E_{ads}$ defined as follows:

$$E_{ads}=E_{T}-E_{SWG}-E_{Cp}-\mu_{M}$$

where $E_{T}$ is the total energy of the half-metallocene of *M* (*M* = Fe, Co, Ni) absorbed SWG; $E_{SWG}$ and $E_{Cp}$ are the energy of pristine SWG and Cp, respectively; and $\mu_{M}$ is the energy of metal ions in the cell.

## 3. Results and Discussion

### 3.1. Adsorption Configurations and Energy

#### 3.1.1. Adsorption of Cp in Stone-Wales Graphene (SWG)

**Figure 2:**
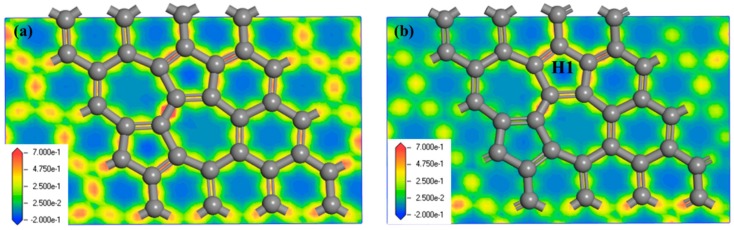
Electron density difference for (**a**) SWG and (**b**) Cyclopentadienyl (Cp)/SWG at the pentagon ring (H1).

To understand how the Cp and half-metallocene are adsorbed in SWG, their adsorption configurations were evaluated and are showed in Figure 1. The adsorption energies and value of charge transfer (*Q_e_*) based on the Mulliken population between SWG and adsorbate corresponding to the different adsorption configurations are listed in Table 1. We discussed the adsorption of Cp on the SWG. The calculated results indicated that Cp absorbed on H1 was the most stable. Meanwhile, the adsorption energy and the smallest distance between Cp and SWG layer is shown in Table 1. The results showed that *E_ads_* varied from −1.09 eV to −1.15 eV and H1 had the highest value (−1.15 eV), which was much larger than that adsorbed in perfect graphene. The large *E_ads_* indicated that the presence of SW defect affected the adsorption process and enhanced the interaction between Cp and the SWG substrate. Figure 2 shows the electron density difference of SWG and Cp/SWG at H1, which indicated that a strong charge transfer process existed between Cp and SWG. *Q_e_* of SWG at H1 was the largest (0.51 e. In fact, the charge distribution was inhomogeneous in SWG. The pentagon ring had a positive charge, whereas the heptagon ring had a negative charge (Figure 2a). Hence, Cp had one electron (i.e., H1) that was absorbed at the site of the pentagon ring. The result was consistent with the absorption energy. 

#### 3.1.2. Adsorption of Half-Metallocene of *M* (*M* = Fe, Co, Ni) in SWG

**Figure 3:**
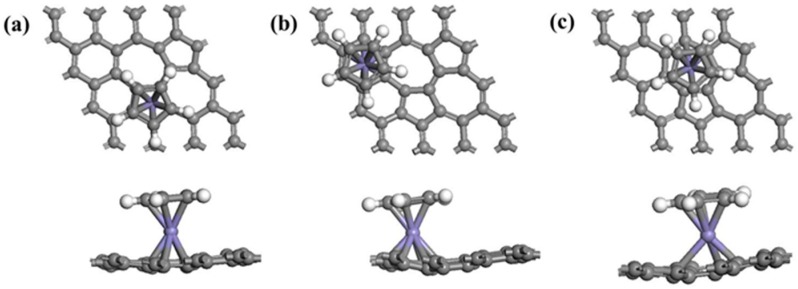
Optimized atomic structures of Cp in SWG at H1 (**a**); hexagon ring (H2) (**b**) and heptagon ring (H3) (**c**).

In this section, we focus on the adsorption of half-metallocene of *M* (*M* = Fe, Co, Ni) in SWG at three different sites, as shown in Figure 3. The parameters describing the adsorption complexes are depicted in Table 1. The value of *E_ads_* ranged from −3.82 eV to −6.23 eV, which was larger than that of SWG. Therefore, metal ions stabilized the adsorption systems of SWG with Cp. The absorption energy had significant differences among different metal ions at the same site. Particularly, SWG-Co-Cp had the strongest *E_ads_*, followed by SWG-Fe-Cp and SWG-Ni-Cp. Meanwhile, for the absorption sites, Cp was located closer to the heptagon ring (H3), suggesting that the metal ions played an important role in the adsorption substrate. *M*^2+^ (*M* = Fe, Co, Ni) with Cp (one electron) had one positive charge, and the heptagon ring had a partial negative charge. Therefore, Cp was located closer to heptagon ring. of SWG at H3 was the largest, which was consistent with its high adsorption energy and value of charge transfer.

### 3.2. Density of States (DOS) of the SWG System

#### 3.2.1. DOS of Cp in SWG

**Figure 4:**
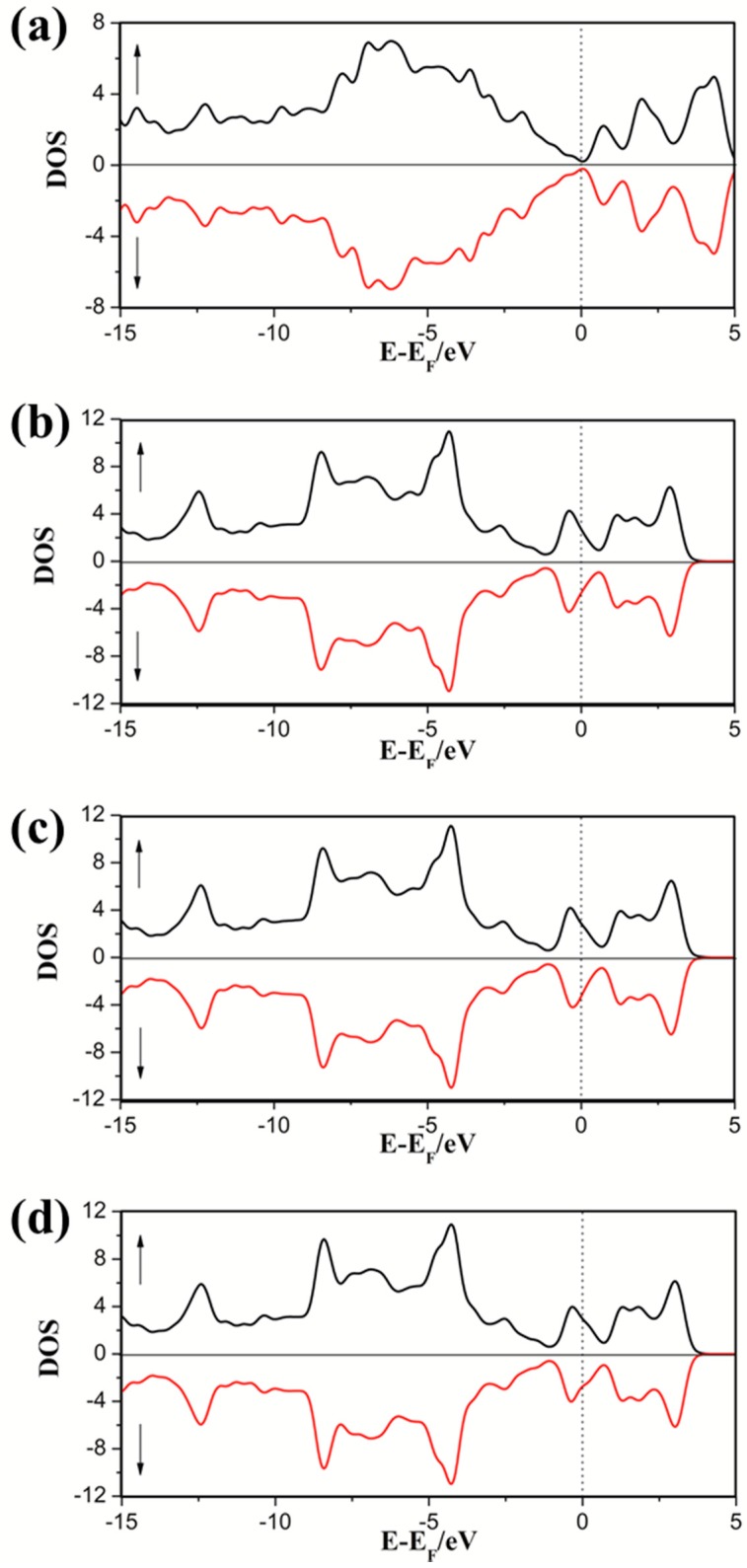
Total electronic density of states (DOS) of pristine SWG (**a**) and Cp on SWG at H1 (**b**); H2 (**c**), and H3 (**d**).

To understand the effect of the electronic properties when Cp absorbed on SWG, the density of states (DOS) of adsorption complexes is illustrated in Figure 4, corresponding to different absorption sites (H1, H2, and H3). The spin-up DOS (majority) and spin-down DOS (minority) were presented in each case, respectively. Pristine SWG is a zero-gap semiconductor in which the Fermi level crossed the Dirac point. In the all cases of absorption configurations, the three systems exhibited metallicity, in which the conduction band passed through the Fermi level. Moreover, the majority and minority DOS were symmetrical, which indicated that their total magnetic moment was zero because of the valence electrons arranged in pairs. From the view point of the molecular orbital, the charge transfer mechanism can be associated with the relative energy positions of the highest occupied molecular orbital (HOMO) and the lowest unoccupied molecular orbital (LUMO) of the adsorbate compared to the SWG Fermi level. If the HOMO is above the Fermi level of SWG, a charge transfer from the adsorbed molecule to SWG may occur. If the LUMO is below the Fermi level, a charge transfer from graphene to the molecule could appear [33]. The HOMO of Cp was 1.68 eV above the Fermi level, deep in the SWG valence band and its LUMO was 7.01 eV above the Fermi level, high in the conduction band of SWG. The HOMO of Cp was the orbital that has a big overlap with the DOS of SWG and thus could cause very big charge transfer value. Therefore, the Cp acted as a very strong donor in the complex systems.

#### 3.2.2. DOS of Half-Metallocene of *M* (*M* = Fe, Co, Ni) in SWG

**Figure 5:**
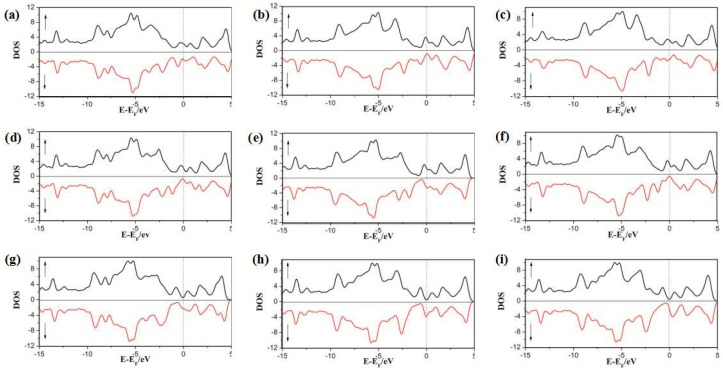
Total electronic DOS of half-metallocene of Fe (**a**–**c**), Co (**d**–**e**), and Ni (**g**–**i**) in SWG at the three hollow sites (H1, H2, and H3).

**Figure 6:**
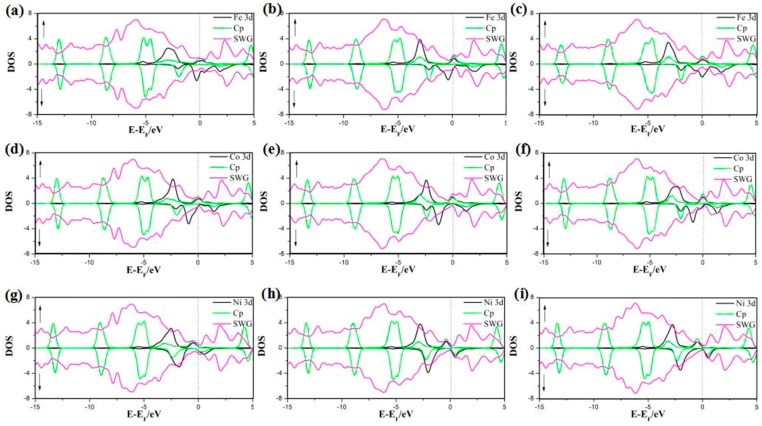
PDOS of half-metallocene of Fe (**a**–**c**), Co (**d**–**e**), and Ni (**g**–**i**) in SWG at the three hollow sites (H1, H2, and H3).

In this part, we focus on the adsorption of half-metallocene of *M* (*M* = Fe, Co, Ni) in SWG at three different sites. The DOS and PDOS are shown in Figure 5 and Figure 6. The results indicated the spin-up and spin-down DOS were asymmetrical, which would generate a magnetic moment. Furthermore, the magnetic moment of all complex systems was tuned by the absorption sites and different metal ions. In Cp/Fe/SWG and Cp/Co/SWG, the spin-up DOS value around the Fermi level was more than that of spin-down DOS. In Cp/Ni/SWG, the spin-up DOS value around the Fermi level was less than that of spin-down DOS. In Cp/Ni/SWG, the spin-up DOS around the Fermi level remained zero. Therefore, the zero-gap semiconductor property of the SWG was maintained in the spin-up channel. On the other hand, the spin-down channel of Cp/Ni/SWG showed a non-zero DOS around the Fermi level and metallicity was correspondingly maintained. The interaction between SWG and Fe ion induced effective shifts between the spin-up and spin-down DOS, which lead to a strong magnetic moment. Considering the strong magnetic property of Fe, Co, and Ni atoms, the spin projected density of states (PDOS) of SWG with half-metallocene of *M* (*M* = Fe, Co, Ni) was showed in Figure 6. The results indicated that the spin DOS of 3D orbital of *M*, Cp, and SWG were all asymmetric between the spin-up and spin-down DOS, which showed that the magnetic property was contributed to by the three parts. The magnetic properties of Cp and SWG were induced from magnetic metal of (*M* = Fe, Co, Ni) by charge transfer. This phenomenon was different from that in a previous study [4].

## 4. Conclusions

The geometrical, energetic, electronic, and magnetic properties of the half-metallocene of *M* (*M* = Fe, Co, Ni) in SWG were investigated using density functional theory (DFT) calculations. The introduction of Cp and half-metallocene increased the conductivity of SWG. Furthermore, the half-metallocene of *M* (*M* = Fe, Co, Ni), with a magnetic behavior, induced different magnetic properties of the adsorption complexes. On the basis of the PDOS results, the magnetic moments of the complex systems were contributed to by the 3D orbital of *M* (*M* = Fe, Co, Ni), molecular orbital of Cp, and SWG. Interestingly, the DOS and magnetic properties were tuned by the absorption sites of Cp and half-metallocene in SWG.

## Figures and Tables

**Table 1:** Summary of results for transition metal (TM) atoms adsorbed in SWG. The properties listed are adsorption energy (*E_ads_*) and the smallest adatom–carbon distance (*d_AC_*).

	*d*	*E*	*Q_e_*
SWG-Cp			
5	3.901	−1.15	0.51
6	3.489	−1.10	0.31
7	3.413	−1.09	0.36
SWG-Fe-Cp			
5	3.692	−5.54	0.38
6	3.384	−5.63	0.54
7	3.603	−5.72	0.58
SWG-Co-Cp			
5	3.653	−6.12	0.38
6	3.383	−6.21	0.44
7	3.456	−6.67	0.48
SWG-Ni-Cp			
5	3.751	−3.82	0.32
6	3.608	−3.83	0.36
7	3.507	−3.85	0.38
